# Fecal steroids, short-chain fatty acids, and microbiota in high- versus low-yielding forest musk deer

**DOI:** 10.1186/s13568-025-01967-6

**Published:** 2025-11-10

**Authors:** Qindan Dai, Jie Wu, Feng Chen, Guimei Jiang

**Affiliations:** Sichuan Institute for Drug Control (Sichuan Testing Center of Medical Devices, Sichuan Institute of Musk Deer Breeding), Chengdu, 611845 China

**Keywords:** Forest musk deer, Steroid hormones, Short-chain fatty acid, Bacterial community, Musk secretion

## Abstract

Adult male forest musk deer (*Moschus berezovskii*; FMD) possess high medicinal and economic value due to their musk secretion capacity. However, under farming conditions, a subset of individuals exhibit abnormal musk secretion or complete secretory failure, resulting in economic losses. This phenomenon is associated with lipid metabolism and gut microbiota alterations. We compared fecal steroid hormones, short-chain fatty acids (SCFAs), and bacterial communities between high-yielding and low-yielding FMD under identical captive conditions. Seven male FMD (2 ~ 6 years old) secreting normal musk comprised the high-yielding group (HFMD), while seven age-matched males secreting abnormal musk formed the low-yielding group (LFMD). Results showed significantly higher concentrations of testosterone (T), estradiol (E2), cortisol, and corticosterone (CORT) in HFMD versus LFMD group (*p* < 0.05). Conversely, SCFA levels showed an inverse pattern: butyrate and hexanoate concentrations were significantly higher in LFMD group (*p* < 0.05). Following multiple testing correction, the relative abundances of *Fibrobacteres* (*p* = 0.013; FDR = 0.170), *Tenericutes* (*p* = 0.013; FDR = 0.107), and *Verrucomicrobia* (*p* = 0.037; FDR = 0.223) were observed to be significantly higher in the HFMD group compared to the LFMD group. Furthermore, distinct microbial biomarkers were identified between the two groups. Certainly, ASV_849 (taxonomically classified as Ruminococcaceae UCG-014) exhibited significant correlations with multiple steroid hormones and SCFAs (*p* < 0.05; FDR = 0.246). Moreover, correlation analysis demonstrated positive associations between musk secretion and E2, T, cortisol, and CORT (*p* < 0.05), but negative correlations with hexanoate (*p* < 0.05). Butyrate and hexanoate were inversely correlated with T (*p* < 0.05). These findings reveal that the gut microbiota may regulate musk secretion through their metabolites (SCFAs), which not only play a key mediating role in microbe-host interactions but may also indirectly influence the process of musk secretion via their interplay with steroid metabolism. This study provides valuable insights into the captive care management of adult male FMD and strategies for enhancing musk secretion.

**Supplementary Information:**

The online version contains supplementary material available at 10.1186/s13568-025-01967-6.

## Introduction

The forest musk deer (*Moschus berezovskii*, FMD) is a small ruminant species belonging to the genus *Moschus*. It inhabits forested regions and is classified as a first-class protected wild animal in China (Li et al. 2016). This species exhibits distinct behavioral traits, including territoriality, solitary habits, and heightened vigilance (Hu et al. 2018a). Male individuals possess a specialized physiological structure called the musk gland, situated between the navel and genitals. In adults, this organ synthesizes, stores, and secretes musk—a highly prized substance in traditional Chinese medicine. Derived from the dried secretions of the male musk deer’s preputial follicles, musk serves as a premium medicinal ingredient with remarkable therapeutic properties, including promoting resuscitation, enhancing blood circulation, and improving collateral flow (Yang et al. 2003; Tang et al. 2018). Consequently, the FMD were heavily hunted in the past for their valuable musk, leading to a dramatic population decline. To protect the species and ensure sustainable musk production, China initiated captive breeding programs in 1958. However, despite the subsequent increase in captive populations, these farmed FMD frequently suffer from health issues, including diarrhea, parasitic infections, and abscesses (Yan et al. 2016; Hu et al. 2018b). Current evidence suggests that these factors not only impede population recovery but also compromise musk yield and quality. While pathological conditions constitute one influential factor, musk secretion is regulated by a complex interplay of biological determinants. Conventionally, aged or physiologically compromised males were thought to be the primary producers of abnormal musk; however, field observations reveal that even healthy young individuals may secrete atypical musk. Notably, clinically asymptomatic adults demonstrate significant variability in both secretion volume and biochemical profiles—ranging from normal musk (brown, solid, and mildly aromatic) to abnormal variants (acrid-smelling white/black secretions or non-secreted musk) (Zhang et al. 2021). Normal musk exhibits a highly complex biochemical composition, primarily consisting of macrocyclic ketones, pyridines, steroids, and other alkaloids, along with polypeptide proteins, fatty acids, esters, and inorganic acids, and atypical musk lacks main biochemical composition, rendering it pharmacologically inactive (Zhang et al. 2021). However, the biosynthetic pathways of these components remain poorly understood, significantly limiting our comprehension of musk formation mechanisms. Current investigations into musk synthesis have employed multiple approaches, including: histological and anatomical studies of the musk gland structure (Qi et al. 2020), analysis of sex hormone regulation in musk secretion (Tang et al. 2018), genetic profiling of musk-related genes (Zhou et al. 2020) and metabolomic analyses of glandular tissue, blood serum, and musk secretions (Wang et al. 2024b). Undoubtedly, accumulating evidence has demonstrated that steroid hormones, particularly sex hormones, serve as critical regulators in the musk formation process (Fan et al. 2018; Tang et al. 2018; Zhou et al. 2019).

Steroid hormones are synthesized from cholesterol and serve as key regulators of lipid metabolism. Cholesterol is acquired through two major pathways: dietary absorption and hepatic de novo biosynthesis (Luo et al. 2020). During digestion, the biliary system secretes cholesterol along with other bile components into the small intestine (Friedman and Nylund 1980). Dietary lipids (e.g., cholesterol) influence gut microbiota composition via microbial-derived metabolites. Research has identified multiple cholesterol-interacting gut bacteria, such as *Bacteroides*, suggesting that diet-dependent mechanisms for altering microbiome-specific cholesterol metabolism (Le et al. 2022). For ruminants, lipid metabolism—associated energy metabolism—is closely intertwined with gut microbiota. Short-chain fatty acids (SCFAs), key microbial metabolites derived from gut microbiota, play pivotal roles in modulating host metabolism by enhancing glucose and lipid homeostasis, regulating energy balance, and exerting immunomodulatory effects through inflammatory response regulation (Agus et al. 2021). Emerging evidence indicates bidirectional interactions between steroid hormones and SCFAs, clinical studies have demonstrated significantly reduced propionate levels in patients with endogenous glucocorticoid excess (Zhang et al. 2023). Consistent with the finding, Qiu et al. (2019a) reported decreased SCFAs production in glucocorticoid-induced obese individuals. Additionally, chemical analysis reveals the presence of ester compounds in musk secretions (Zhang et al. 2021). Based on the above findings, it can be inferred that musk secretion in FMD is associated with SCFAs and lipid metabolism. To date, there have been limited investigations into the potential influence of gut microbiota and SCFAs metabolism on musk secretion. Our review of existing literature reveals that hormonal regulation plays a significant role in FMD musk secretion behavior. However, critical knowledge gaps remain regarding the differential patterns of steroid hormones, SCFAs, and gut microbial composition between FMD exhibiting normal versus abnormal musk secretion patterns, particularly under identical dietary conditions.

Building upon the well-established correlation between steroid hormones and lipid metabolism, and considering the distinctive microbial metabolic system of ruminants, we hypothesized that musk secretion in FMD is regulated through an integrated mechanism involving not only hormonal control but also SCFAs associated with lipid metabolism pathways and gut microbial modulation. To test this hypothesis, we conducted a comparative analysis of fecal steroid hormones, SCFAs, and gut microbial composition in FMD maintained under identical dietary conditions, examining their potential associations with musk production efficiency. These findings provide crucial insights into the synergistic roles of steroid hormones, SCFAs, and gut microbiota in modulating musk secretion. Furthermore, This study investigates the differences between high- and low-yielding FMD, providing an experimental foundation for developing strategies to improve musk secretion in low-yielding individuals.

## Materials and methods

### Animals and feeding management

This study utilized a total of 14 adult FMD, aged 2–6 years, from the Markon Musk Deer Farm at the Sichuan Institute of Musk Deer Breeding in Aba Prefecture, Sichuan Province, China. All subjects were housed individually under standardized conditions, with clean enclosures maintained. The FMD received a consistent diet consisting of leaves provided at 6:00 AM and a supplemental mixture administered at 16:00 PM daily. This nutritional supplement contained a blend of concentrate and fresh vegetables, including carrot, lettuce, pumpkin, and cabbage. Water was available ad libitum.

**Table 1:** Characteristics of forest musk deer and their musk secretions in the present study

Samples	Age (years)	Musk description	
		Musk appearance	Musk secretion (g)
HFMD-1	3	NM	22.6
HFMD-2	2	NM	11.4
HFMD-3	2	NM	14.1
HFMD-4	4	NM	12.0
HFMD-5	4	NM	14.7
HFMD-6	2	NM	10.5
HFMD-7	2	NM	11.8
LFMD-1	2	EM	0.0
LFMD-2	4	WM	0.0
LFMD-3	4	WM	0.0
LFMD-4	2	EM	0.0
LFMD-5	2	EM	0.0
LFMD-6	6	WM	0.0
LFMD-7	6	WM	0.0

NM, normal musk: standard musk with rich aroma; WM, white musk: white musk with no fragrance; EM, empty musk: musk pod without secretion. WM and EM are deemed valueless, with a recorded secretion of zero

### Experimental design and sample collection

A total of 14 musk deer were assigned to two experimental groups: the HFMD group (*n* = 7), consisting of individuals secreting normal, high-quality musk, and the LFMD group (*n* = 7), comprising animals producing either low-quality musk or complete secretory failure. The musk extraction operation was performed in September 2024. And key traits of FMD and their musk are summarized in Table 1. To minimize potential stress-induced disturbances from musk extraction operation, fecal samples were collected in November 2024. Fresh feces from each FMD were collected in sterile tubes prior to morning feeding and immediately stored at − 80 °C for subsequent analyses.

### Determination of fecal steroid hormone

Fecal samples (1 g wet weight) were homogenized in 15 mL sterile centrifuge tubes with 9 mL of phosphate-buffered saline (PBS; 0.01 mol/L, pH 7.2–7.4) by vortexing for 1 min. The homogenate was then centrifuged (5000 rpm, 4 °C, 15 min), and the supernatant was collected and stored at − 20 °C until analysis. Given the phylogenetic proximity of FMD to bovids (Chen et al. 2019), fecal testosterone (T), estradiol (E2), cortisol, and corticosterone (CORT) concentrations were quantified using commercial bovine ELISA kits (Jiangsu Baolai Biotechnology Co., Ltd, Taizhou, Jiangsu China). Each sample was analyzed in triplicate to ensure data reliability.

### Short-chain fatty acid

Approximately 50 mg of sample was homogenized with 80% methanol-water solution using a tissue grinder. After centrifugation (15,000 rpm, 4 °C, 15 min), 20 µL of supernatant was transferred to a 1.5 mL microcentrifuge tube. The extract was then derivatized by adding EDC solution and 3-NPH reagent. The reaction mixture was diluted to 500 µL with initial mobile phase solution, vortex-mixed thoroughly, and 200 µL of the final solution was transferred to an autosampler vial for LC-MS/MS analysis.

The SCFA analysis was performed using an AB Sciex 4500 triple quadrupole mass spectrometer (Sciex, Framingham, MA, USA) coupled with a Jasper HPLC system. Chromatographic separation was achieved on an Agilent Poroshell 120 EC-C18 column (2.1 × 100 mm, 2.7 μm) maintained at 40 °C, with a 2 µL injection volume. The mobile phase consisted of water and a 1:1 (v/v) methanol: acetonitrile mixture. Mass spectrometric detection was conducted in negative ionization mode using multiple reaction monitoring (MRM) for optimal sensitivity and selectivity. The system was calibrated with authentic standards including acetic acid, propionic acid, butyric acid, isobutyric acid, valeric acid, isovaleric acid, and hexanoic acid, with all solvents being LC-MS grade and water purified through a Milli-Q system.

### DNA extraction and 16 S rRNA gene sequencing

Genomic DNA (gDNA) was extracted from fecal samples using the Zymo Research BIOMICS DNA Microprep Kit (Zymo Research, Irvine, CA, USA). DNA integrity was verified by 0.8% agarose gel electrophoresis, while concentration and purity (A260/A280 and A260/A230 ratios) were quantified using a TECAN F200 microplate reader (Tecan, Shanghai, China). The V4 hypervariable region of the bacterial 16 S rRNA gene was amplified via PCR (Applied Biosystems^®^ 9700 PCR System, Thermo Fisher Scientific Inc., Waltham, MA, USA) using universal primers 515 F (5′-GTGYCAGCMGCCGCGGTAA-3′) and 806R (5′-GGACTACHVGGGTWTCTAAT-3′). Equimolar amounts of PCR products from all samples were pooled for downstream analysis. PCR amplicons were fragmented by sonication and processed into sequencing libraries using the NEBNext Ultra DNA Library Prep Kit for Illumina (New England Biolabs, Beijing, China), following the manufacturer’s protocol with dual-index barcoding. Libraries were prepared with the NEBNext Ultra II DNA Library Prep Kit for Illumina (New England BioLabs, MA, USA). Paired-end sequencing (PE250) was performed on an Illumina NovaSeq 6000 instrument using the NovaSeq 6000 SP Reagent Kit V1.5 (Illumina, San Diego, CA, USA). Raw sequencing reads underwent quality control and filtering to ensure high-quality data for subsequent bioinformatic analysis.

### Sequencing data analysis

Paired-end reads were merged using FLASH (version 1.2.11) (Magoč and Salzberg 2011). Demultiplexing based on sample-specific barcodes was performed with sabre, after which barcode sequences were trimmed. Subsequent quality filtering, denoising, and chimera removal were conducted in QIIME2 (version 2020.2) (Bolyenet al. 2019) using the Deblur algorithm, yielding a table of amplicon sequence variants (ASVs) and representative sequences. Taxonomy was assigned to ASVs using a naïve Bayes classifier pre-trained on the SILVA (version 138) (Quast et al. 2013) reference database. The rarefaction curves were generated based on the number of ASVs. Additionally, relative abundance normalization was applied by dividing the count of each feature by the total read count of the respective sample, resulting in proportional abundance data used in downstream ecological analyses and visualization. All sequence data processing was conducted in R software (version 4.5.1). Microbial community diversity was evaluated using both alpha and beta diversity metrics computed through the Vegan package (Oksanen et al. 2016). Specifically, inter-sample community dissimilarity was quantified using Bray-Curtis distances generated by the vegdist function. For multivariate visualization, principal coordinates analysis (PCoA) was performed using the ape package (Paradis et al. 2004), with Bray-Curtis dissimilarity matrices as input. Statistical significance of observed group differences was assessed through permutational multivariate analysis of variance (PerMANOVA) using Vegan’s adonis function. To identify microbial taxa and metabolites exhibiting differential abundance and association, we performed multivariate association analysis using microbiome multivariable associations with linear models (MaAsLin2, version 1.22.0) (Mallick et al. 2021). The analysis incorporated a microbial abundance table encompassing taxa at the phylum, genus, and ASV levels, along with metabolite abundances and metadata that included the group variable and adjusted for covariates (age). Linear models (LM) were used as the analytical framework. Raw microbial read counts were normalized via total sum scaling (TSS) and subsequently log10-transformed. The analysis was performed using the following default parameter settings: normalization = “TSS”, transform = “log”, and analysis method = “LM”. To control for multiple testing, the Benjamini–Hochberg (BH) procedure was applied for false discovery rate (FDR) correction. Owing to the exploratory nature and limited sample size of this study, a relaxed false discovery rate (FDR) threshold (FDR < 0.3) was employed to identify potentially differential features warranting further validation. Effect sizes (coefficient) and standard errors were estimated for each association. Results were filtered to retain associations meeting either a FDR < 0.3 or a nominal *p* < 0.05, along with an absolute effect size greater than zero. Statistically significant associations were visualized using a heatmap. In addition, relative abundance bar plots were generated for phylum- and genus-level taxa, while volcano plots and bar charts were used to visualize ASV level results. To complement MaAsLin2, which strictly controls the false discovery rate (FDR < 0.3) but may overlook certain subtle yet biologically relevant associations, we also performed linear discriminant analysis effect size (LEfSe) using the online LEfSe platform (https://huttenhower.sph.harvard.edu/galaxy/), applying a threshold of LDA score > 2.0. LEfSe uses linear discriminant analysis to assess effect size and identify group-specific microbial features that could be overlooked by MaAsLin2. The combined approach offers a more comprehensive view of microbial differences between groups. The results of the LEfSe analysis are visualized through cladograms and effect size bar plots to illustrate phylogenetic relationships and highlight statistically significant taxa.

### Statistical analysis

Fecal steroid hormones, volatile fatty acids, and alpha-diversity indices—was evaluated using the Shapiro–Wilk test. Data that followed a normal distribution were compared between groups using independent samples t-tests, following verification of homogeneity of variances with Levene’s test. In cases where data were normally distributed but variances were unequal, Welch’s t-test was applied. For data that did not meet the normality assumption, the non-parametric Mann–Whitney U test was used. All statistical analyses were performed using SPSS (version 27.0 for Windows; SPSS Inc., Chicago, USA). Results are expressed as mean ± standard error of the mean (SEM). To evaluate the associations among steroids, short-chain fatty acids, and musk yield, Pearson correlation analysis was conducted. The results were visualized as a heatmap generated using Origin 2022b (OriginLab Corp., MA, USA). Statistical significance was set at *p* < 0.05.

## Results

### Steroid hormone

**Table 2:** The differences of steroid hormone content between the HFMD and LFMD groups

Items	Groups		SEM	*p* value
	HFMD	LFMD		
E2 (pg/g)	684.55	410.71	39.932	< 0.001
T (pg/g)	2033.54	1394.48	106.193	< 0.001
Cortisol (ng/g)	16.15	10.08	0.955	< 0.001
CORT (ng/g)	2194.47	1372.20	119.585	< 0.001

E2, estradiol; T, testosterone; CORT, corticosterone

Obviously, the HFMD group exhibited significantly higher (*p* < 0.001) levels of E2, T, cortisol, and CORT compared to the LFMD group (Table 2).

### Fecal Short-chain fatty acid

**Table 3:** The differences of fecal short-chain fatty acid between the HFMD and LFMD groups

Items (µg/g)	Groups		SEM	*p* value
	HFMD	LFMD		
Total SCFAs	3028.31	3742.28	332.130	0.298
Acetate	2050.00	2305.71	201.596	0.547
Propionate	397.29	476.86	56.782	0.506
Butyrate	239.00	415.57	41.748	0.040
Isobutyrate	54.80	66.66	5.813	0.327
Isovalerate	33.21	39.21	3.758	0.447
Valerate	37.13	57.13	6.013	0.097
Hexanoate	2.95	4.71	0.419	0.028
A:P	6.18	5.14	0.696	0.479

The total SCFAs concentration is calculated as the sum of all individual SCFA; A:P, acetate/propionate ratio

As illustrated in Table 3, no significant difference (*p* > 0.05) of total SCFAs, acetate, propionate, isobutyrate, isovalerate and valerate concentrations were observed between the two groups. Similarly, acetate/propionate (A: P) did not show obvious difference (*p* > 0.05). The LFMD group exhibited a higher butyrate and hexanoate concentrations when compared to HFMD group (*p* < 0.05).

### Microbial data acquisition and analysis

**Fig. 1:**
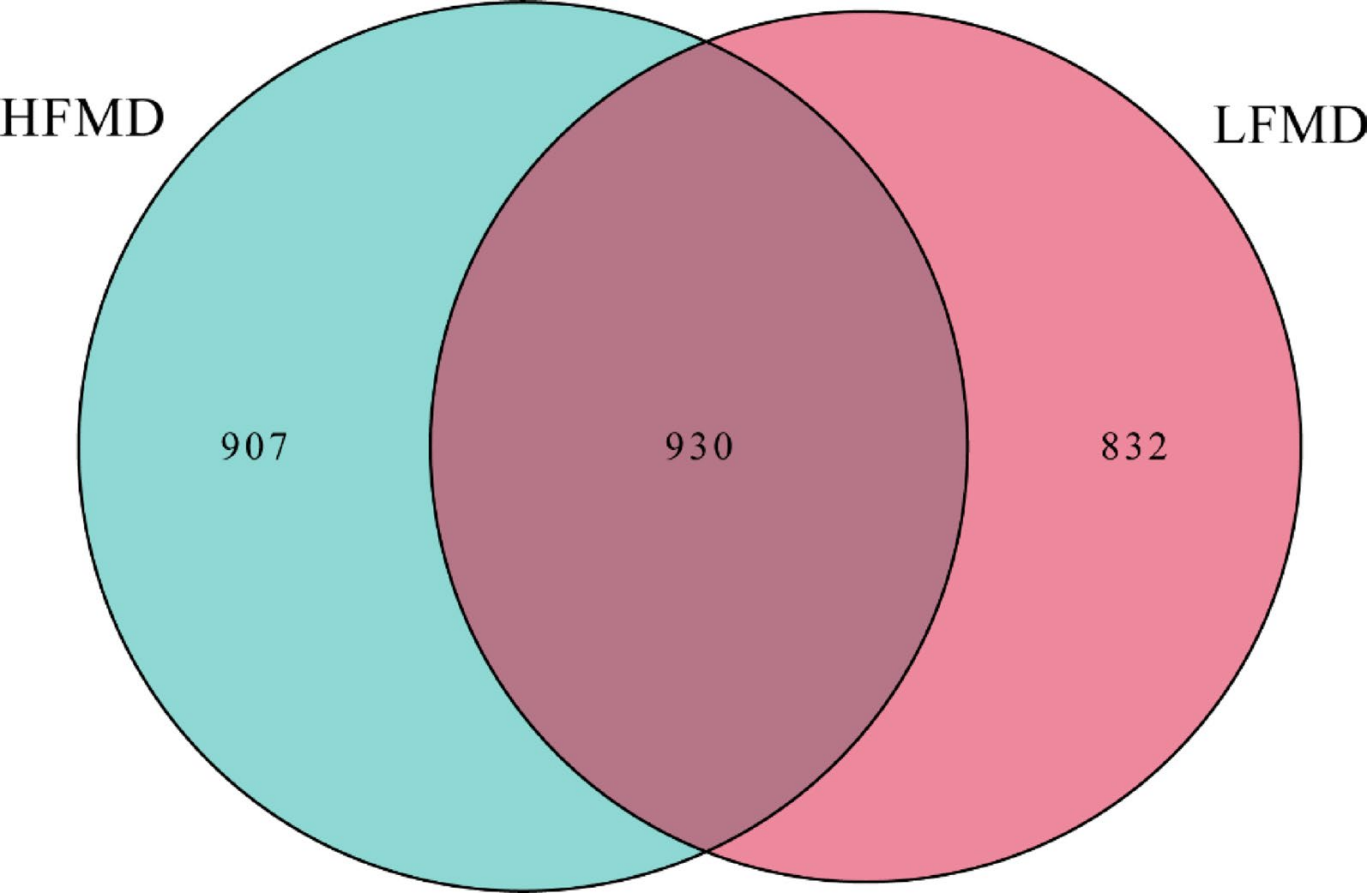
Venn diagram illustrating the comparison between the HFMD and LFMD groups. The green circle denotes the HFMD group, while the red circle corresponds to the LFMD group

In the current study, 14 fecal samples were collected from the two groups. We obtained a total of 769,831 raw sequences by 16 S rRNA gene sequencing, with an average of 54,988 ± 828 (mean ± standard error) per sample (Supplementary Table S1). After quality filtering of sequence, the effective sequences were obtained, with an average of 53,088 ± 793 per sample. the Venn diagram (Fig. 1) illustrates the distribution of features between the HFMD and LFMD groups. To evaluate sequencing quality and the adequacy of sequencing depth, Q30 values (Supplementary Table S1) and rarefaction curves (Supplementary Fig. S1) were generated for each sample. All rarefaction curves reached a clear plateau, demonstrating that sufficient depth was achieved to reliably characterize the fecal bacterial community. Specifically, the HFMD group has 907 unique ASVs, while the LFMD group has 832 unique ASVs. Additionally, there are 930 shared ASVs between the two groups.

### Bacterial alpha- and beta-diversity diversity analysis

**Fig. 2:**
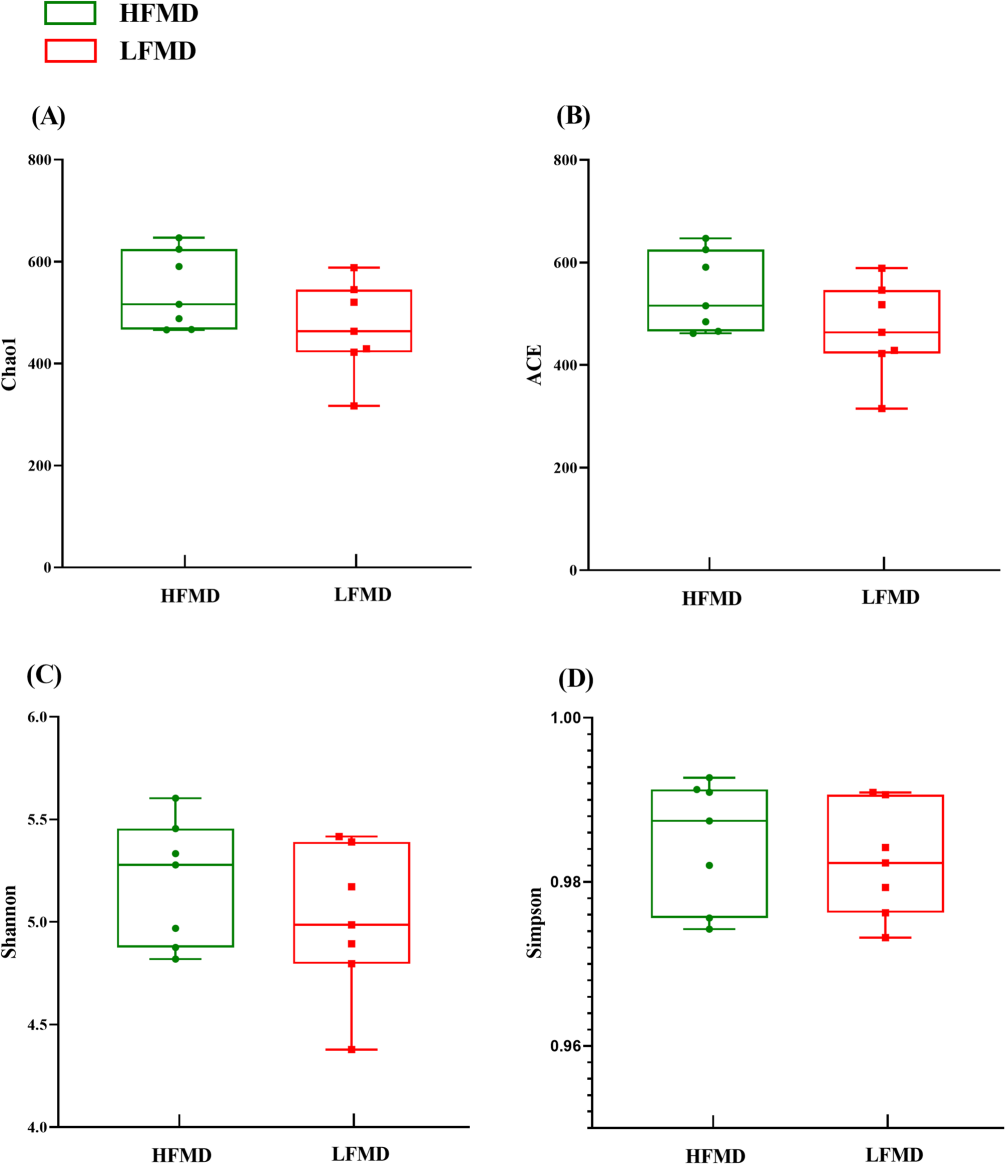
α diversity indices of gut microbiota. **A** Chao1, **B** ACE, **C** Shannon, and **D** Simpson. Each dot (●) represents a single biological sample from the HFMD group; each square (■) represents a sample from the LFMD group, with the x-axis indicating the experimental groups and the y-axis representing the calculated index values. Group distributions are illustrated as boxplots, where red and green boxes denote the HFMD and LFMD groups, respectively

**Fig. 3:**
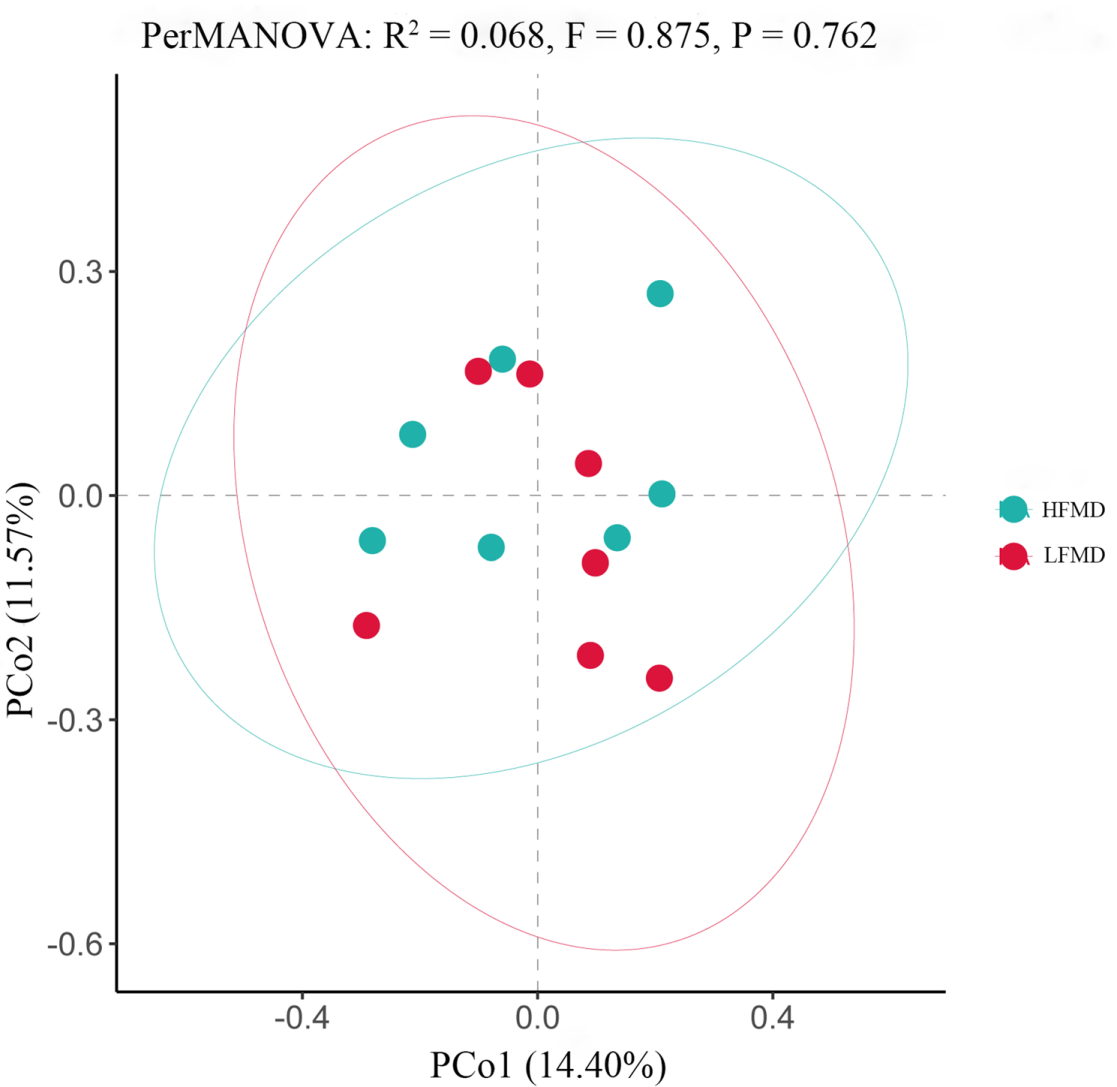
Principal coordinates analysis of bacterial communities between the HFMD and LFMD groups based on the Bray–Curtis distance. Each point in the graph represents one sample, and the green circles represent the HFMD group, while the red circles denote the LFMD group. The distance between points represents the level of difference

Alpha-diversity showed that the Chao1, ACE, Shannon and Simpson indexes were similar (*p* > 0.05) between the HFMD and LFMD groups (Fig. 2). In this study, the PCoA analysis (Fig. 3) based on Bray-Curtis dissimilarity matrices revealed no clear separation in fecal microbiota structure between the HFMD and LFMD groups. Meanwhile, the PerMANOVA analysis of inter-group distances showed no significant microbial community differences between the two groups (R^2^ = 0.068, *p* = 0.875).

### Analysis of differences between the HFMD and LFMD gut microbiota

**Fig. 4:**
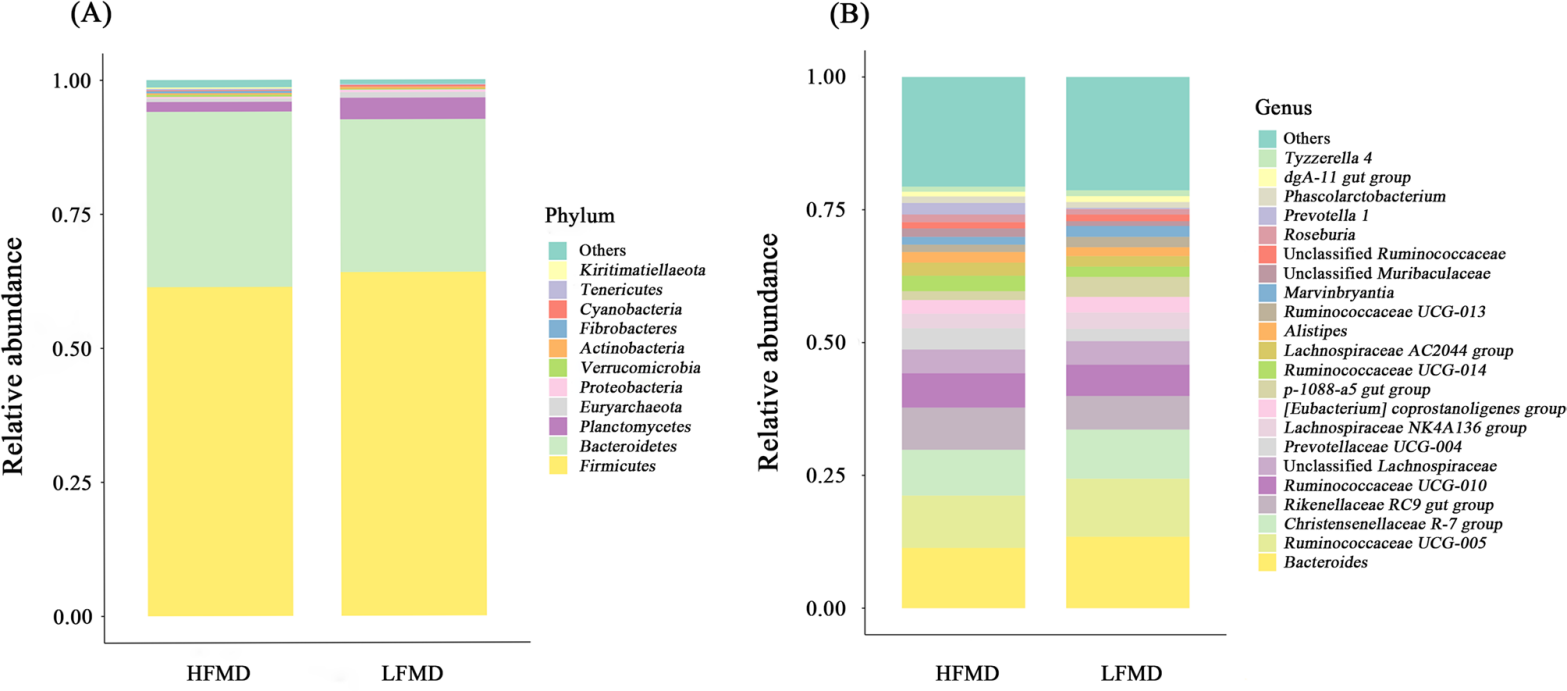
Histogram of relative abundance. The x-axis represents groups and the y-axis represents relative abundance. **A** Phyla exhibiting relative abundances greater than 0.5%. **B** Genera exhibiting relative abundances greater than 1%. Other species were combined as “Others”

At the phylum level, a total of 22 phyla were observed in 16 samples, of which *Firmicutes* (HFMD = 61.38% and LFMD = 64.11%), *Bacteroidetes* (HFMD = 32.69% and LFMD = 28.49%), and *Planctomycetes* (HFMD = 1.85% and LFMD = 4.01%) were the most abundant (Fig. 4A and Supplementary Table S2). The relative abuncances of *Firmicutes*, *Bacteroidetes*, *Planctomycetes*, *Spirochaetes*, *Euryarchaeota* and *Proteobacteria* were similar (*p* > 0.05; FDR > 0.30) between HFMD and LFMD groups. Furthermore, a similar (*p* > 0.05; FDR > 0.30) *Firmicutes*/*Bacteroidetes* ratio (F: B) was recorded in HFMD group compared to LFMD group. Then, compared with the LFMD group, the HFMD group exhibited a significantly higher relative abundance of *Fibrobacteres* (*p* = 0.013; FDR = 0.170), *Tenericutes* (*p* = 0.013; FDR = 0.107) and *Verrucomicrobia* (*p* = 0.037; FDR = 0.223).

At the Genus level, 256 genera were obtained, results showed that the predominant genera in the two groups mainly included *Bacteroides* (HFMD = 11.37% and LFMD = 13.46%), *Ruminococcaceae UCG-005* (HFMD = 9.84% and LFMD = 10.91%), *Christensenellaceae R-7 group* (HFMD = 8.61% and LFMD = 9.25%), *Rikenellaceae RC9 gut group* (HFMD = 7.94% and LFMD = 6.31%) and *Ruminococcaceae UCG-010* (HFMD = 6.48% and LFMD = 5.89%) (Fig. 4B and Supplementary Table S3). Whereas genera with a relative abundance exceeding 1%, no significant differences (*p* > 0.05; FDR > 0.3) were observed between the two groups.

**Fig. 5:**
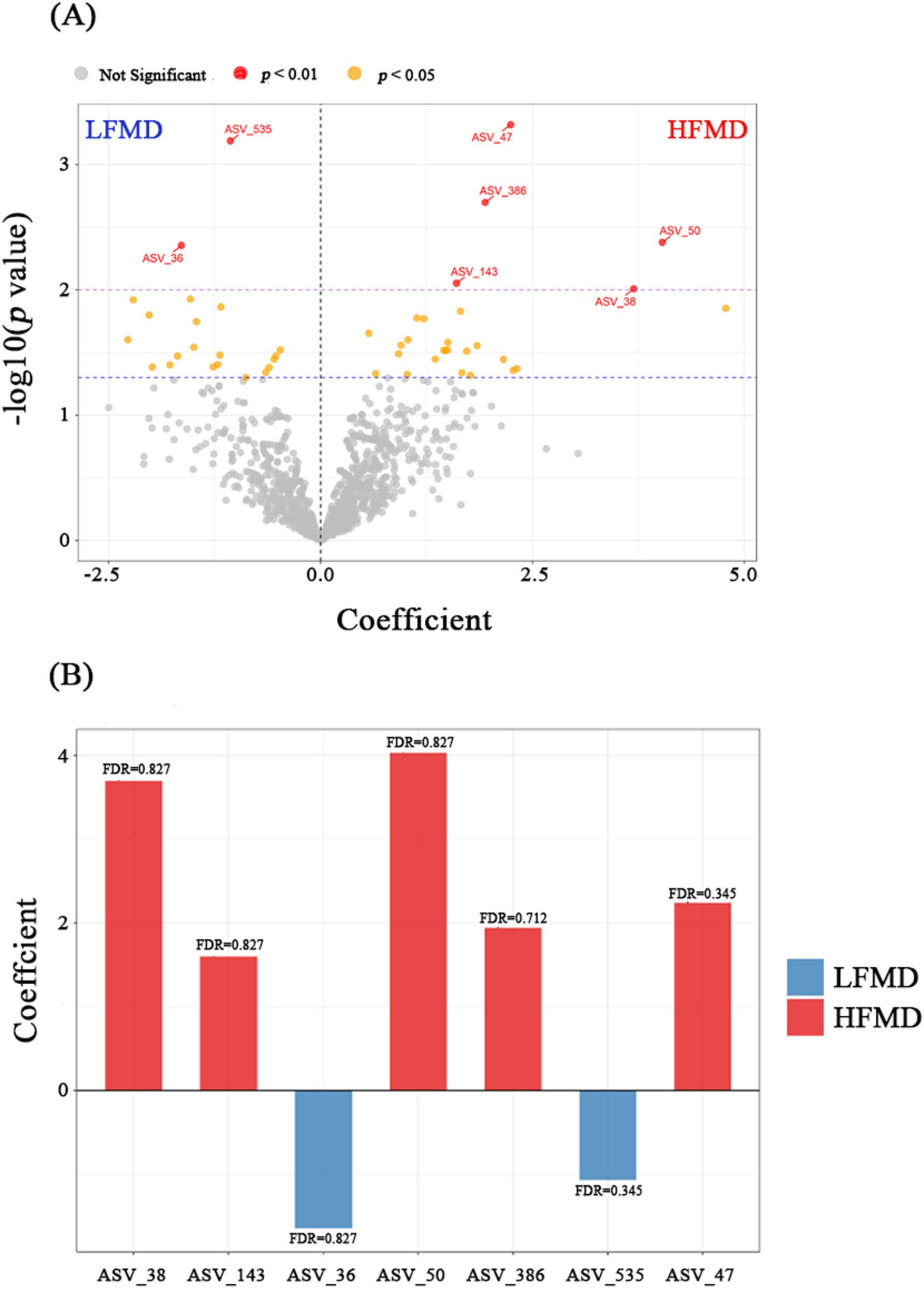
Differential analysis of microbial communities between the HFMD and LFMD groups at the ASV level. **A** Volcano plot of differential features: orange points represent taxa with *p* < 0.05, and red points indicate those with *p* < 0.01. **B** Bar plot showing significantly different taxa (*p* < 0.01) at the genus level. Taxonomic assignments are as follows: ASV_38, *Prevotellaceae UCG-004*; ASV_143, *Rikenellaceae RC9 gut group*; ASV_50, *Rikenellaceae RC9 gut group*; ASV_535, *Christensenellaceae R-7 group*; ASV_47, *Rikenellaceae RC9 gut group*; ASV_386, *Ruminococcaceae UCG-010*; ASV_36, *Incertae Sedis*

The results of the differential analysis at a more refined ASV level are shown in Fig. 5. The volcano plot indicated a greater number of ASVs with nominally differential abundance (*p* < 0.05, FDR > 0.3) in the HFMD group compared to the LFMD group (Fig. 5A). A subset of ASVs showing the most pronounced nominal differences (*p* < 0.01, FDR > 0.3) was further examined using a bar chart (Fig. 5B). Among these, ASV_38, ASV_143, ASV_50, ASV_386, and ASV_47 exhibited markedly higher abundance in the HFMD group under this uncorrected significance threshold. Similarly, ASV_36 and ASV_535 also demonstrated increased abundance in the LFMD group, though none of these associations remained significant after correction for multiple testing.

**Fig. 6:**
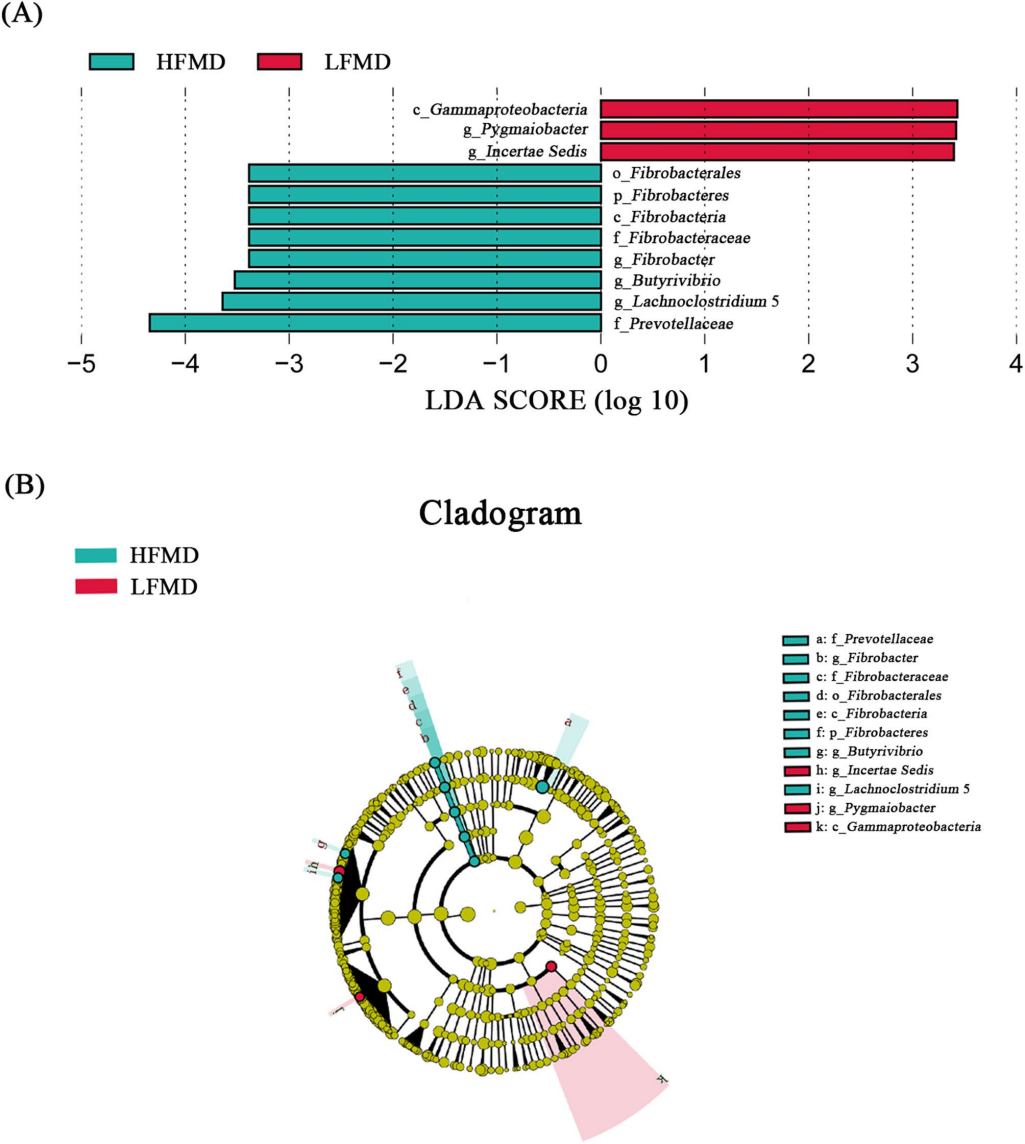
LEfSe analysis. **A** Plot from LEfSe analysis. The plot was generated using the online LEfSe project. The length of the bar column represents the LDA score. The figure shows the microbial taxa with significant differences between the HFMD (green) and LFMD (red) groups (LDA score > 2). **B** A cladogram showing the differences in relative abundance of taxa at five levels between the HFMD and LFMD groups. The plot was generated using the online LEfSe project. The green and red circles mean that HFMD and LFMD groups showed differences in relative abundance and yellow circles mean non-significant differences

As demonstrated in Fig. 6, phylogenetic and taxonomic differences between the two groups were visualized through a cladogram and further quantified by LEfSe analysis. Notably, plot from LEfSe analysis (Fig. 6A) display LDA scores of microbial taxa with significant differences, and the cladogram (Fig. 6B) showed differences in 11 taxa between the HFMD and LFMD groups. At the genus level, significant microbial biomarkers for the HFMD group included *Fibrobacter*, *Butyrivibrio* and *Lachnoclostridium 5*, while the LFMD group showed distinct biomarkers comprising *Pygmaiobacter* and *Incertae Sedis*.

### Correlation analysis

**Fig. 7:**
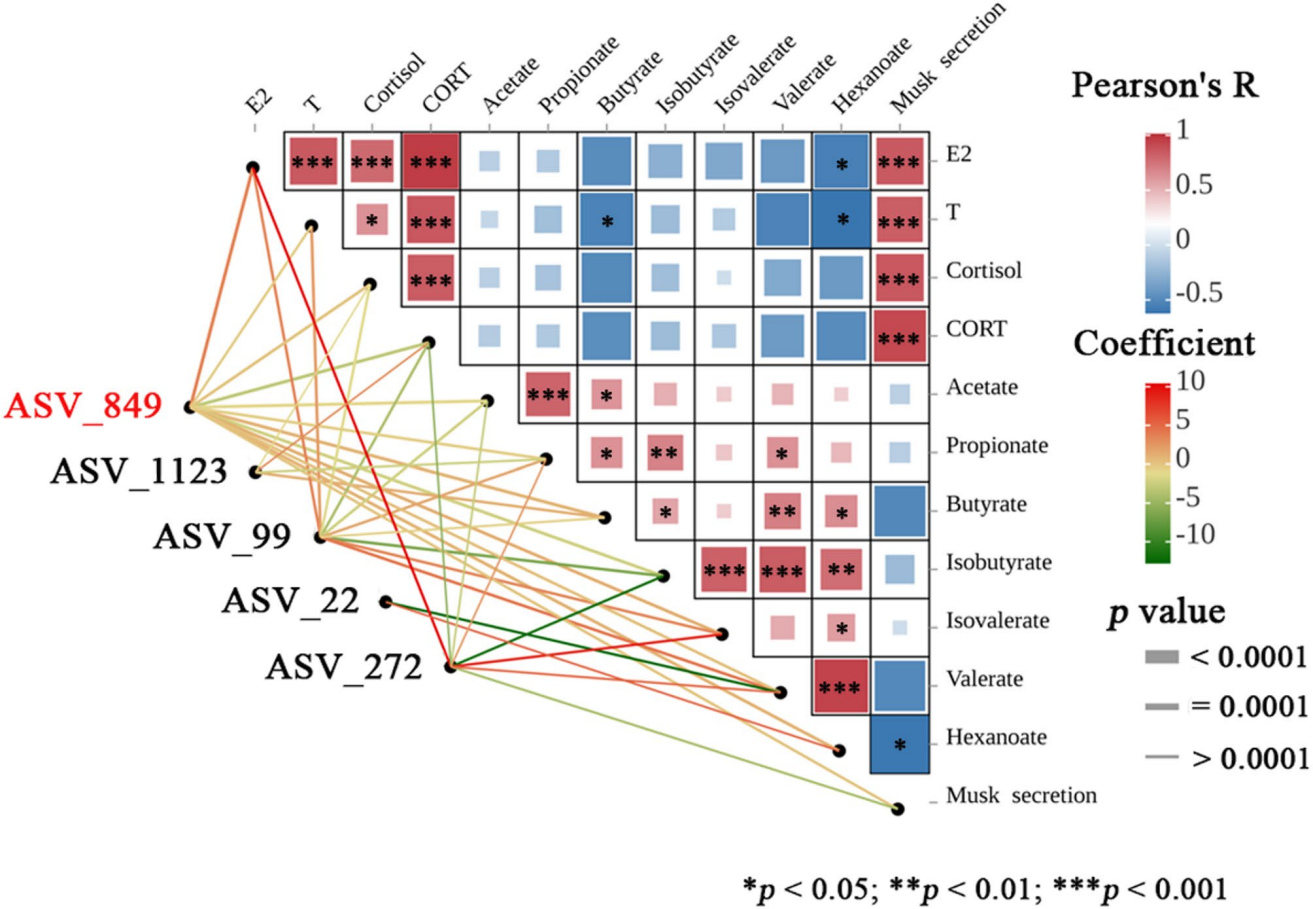
Integrated correlation network of gut microbiota (ASV level) and key metabolic parameters. Links show significant relationships between microbial features and environmental variables (MaAsLin2) and among environmental variables (Pearson). Red/blue colors indicate positive/negative correlations respectively, with color intensity reflecting correlation strength. Only ASVs with a false discovery rate (FDR) below 0.5 are displayed. Selected taxonomic assignments are shown: ASV_849, *Ruminococcaceae UCG-014*; ASV_1123, *Desulfovibrio*; ASV_99, *Christensenellaceae R-7 group*; ASV_22, *Bacteroides*; ASV_272, *Ruminiclostridium 6*

As illustrated in Fig. 7, the association analysis identified five ASVs with an FDR threshold below 0.5 that were significantly correlated with physicochemical parameters. Notably, ASV_849 demonstrated significantly positive associations with E2, butyrate, isovalerate, and hexanoate (*p* < 0.001; FDR = 0.246), and significantly negative associations with CORT, acetate, and isobutyrate (*p* < 0.001; FDR = 0.246). In addition, ASV_849 was tentatively positively associated with musk secretion (*p* < 0.001, FDR = 0.332), whereas ASV_272 showed a potential negative association (*p* < 0.001, FDR = 0.396).

Pearson correlation analysis revealed significant positive correlations between musk secretion and all measured hormones: E2 (*R* = 0.84, *p* < 0.001), T (*R* = 0.82, *p* < 0.01), Cortisol (*R* = 0.85, *p* < 0.001), and CORT (*R* = 0.91, *p* < 0.001). Conversely, butyrate (*R* = −0.54, *p* < 0.05) and hexanoate (*R* = -0.61, *p* < 0.05) concentrations were significantly inversely associated with T. Furthermore, hexanoate concentration exhibited significant negative correlations with both E2 (*R* = −0.55, *p* < 0.05) and musk secretion (*R* = -0.60, *p* < 0.05).

## Discussion

In commercial musk secretion systems, both the quality and yield of musk secretions from captive FMD greatly influence overall production efficiency. Previous studies indicate that musk quality is determined by multiple interrelated factors, such as hormonal regulation, genetic predisposition, and disease susceptibility (Yan et al. 2016; Fan et al. 2018; Zhou et al. 2019). The quality of musk can be preliminarily assessed based on its color and physical characteristics. For instance, abnormal musk secretions—such as white or black discoloration accompanied by a pungent odor, as well as hollow musk sac glands (Zhang et al. 2021). In the present study, the HFMD group exhibited significantly higher levels of T, E2, cortisol, and CORT compared to the LFMD group, consistent with prior research (Wang et al. 2024a) indicating that high musk-yielding FMD possess elevated T levels. Similarly, the observed positive correlation between steroid hormone levels and musk secretion provides direct evidence of endocrine regulation in this process. Interestingly, one study found that T and E2 appear to play a predominant role in shaping musk composition specifically during the initial secretion phase, rather than the subsequent maturation stage (Fan et al. 2018). Steroid hormones play a crucial role in musk secretion, as evidenced by multiple studies. Yang et al. (2021b) established that the musk gland of male FMD exhibits endogenous steroid hormone biosynthesis capability, with musk secretions containing diverse steroid classes including androgens, progestins, estrogens, and sterols. This finding is further supported by Zhang et al. (2021), who reported substantially reduced concentrations of both steroid hormones and amino acids in white musk relative to normal musk specimens. Thus, steroid compounds play a dual regulatory role, not only mediating musk secretion processes but also determining the characteristic chemical profile of musk. T and E2, the primary sex steroids governing gonadal development, are detectable in both sexes of musk deer with concentrations exhibiting marked fluctuations across developmental stages (Russell andGrossmann 2019). Concurrently, cortisol and CORT serve as pivotal homeostatic mediators, orchestrating metabolic and endocrine adaptive responses (Vandewalle et al. 2018; Escoter-Torres et al. 2019). Chronister et al. (2021) demonstrated that elevated circulating levels of cortisol, E2, and T correlated with heightened susceptibility to depression- and anxiety-like behaviors. Their findings further identified interactive effects between T and cortisol in regulating neuroendocrine-emotional pathways. In light of these findings, steroid hormones serve as key regulators of musk secretion, demonstrating significant positive associations with musk secretion in FMD. Notably, individuals with high musk productivity appear to display heightened sensitivity to emotional stressors. The underlying mechanisms remain unclear: whether these physiological responses stem from endogenous neuroendocrine pathways, are mediated by exogenous environmental triggers that disrupt hormonal homeostasis, or involve a combination of both.

Steroid hormones also play a crucial role in regulating lipid metabolism. T deficiency has been shown to exacerbate diet-induced hepatic lipid accumulation, while normal T levels may help regulate adipose tissue mass by stimulating lipid oxidation (Gibney et al. 2005; Nikolaenko et al. 2014). As a ruminant species, the FMD ferments structural and non-structural carbohydrates in the rumen through microbial activity, producing SCFAs. These SCFAs are rapidly absorbed by the ruminal epithelium and can supply up to 75% of the total metabolizable energy (Siciliano-Jones and Murphy 1989; Dijkstra et al. 2005; He et al. 2018), while the hindgut contributes an additional 12% of the energy supply (Wu et al. 2023). SCFAs function both as metabolic energy sources and signaling molecules, regulating hormone secretion, anti-inflammatory responses, apoptosis, and lipid metabolism (Farzi et al. 2015; Schönfeld and Wojtczak 2016). Musk secretion is regulated by steroid hormones, and during the musk secretion period, steroid hormone levels rise sharply, accompanied by testicular swelling and fasting behavior in FMD. Accordingly, during the fasting period, SCFAs become particularly crucial as the primary energy supply for ruminants. Currently, there is limited research investigating SCFA concentrations in FMD feces, and the proportional distribution of SCFAs within the musk deer intestinal tract remains unclear. Our findings reveal substantial variations in SCFA content, with acetate concentrations ranging from 1,100 to 3,680 µg/g, propionate from 183 to 841 µg/g, and butyrate from 181 to 657 µg/g. These results demonstrate considerable fluctuation in SCFA levels across samples. The FMD exhibits a significantly higher average A: P ratio compared to other ruminants (Qiu et al. 2019b). This elevated A: P ratio suggests enhanced cellulose digestibility (Zhou et al. 2017), which may reflect evolutionary adaptations to their natural diet. The observed ratio could be attributed to the species’ selective foraging behavior, particularly their preference for young leaves that typically contain higher cellulose content and lower lignin compared to mature foliage. In our study, the LFMD group exhibited elevated butyrate and hexanoate in feces versus HFMD group. Butyrate serves multiple physiological functions as the preferred energy substrate for colonocytes while also mitigating oxidative stress, promoting gastrointestinal development, and modulating gut microbiota composition (Niwińska et al. 2017; Bedford and Gong 2018; Liu et al. 2021). Although hexanoate is produced in substantially lower quantities compared to the predominant SCFAs (acetate, propionate, and butyrate), emerging evidence suggests its potential beneficial effects on hepatic lipid metabolism and insulin sensitivity (Akpa et al. 2010; Rial et al. 2018). Evidence suggests that mobilization of cholesterol into steroidogenic pathways is regarded as the rate-limiting step in the rapid production of steroids (Miller and Auchus 2001). Butyrate can reduce serum triglyceride, inhibits cholesterol biosynthesis in vitro (Gao et al. 2009; Zhang et al. 2017), failing to enhance hepatic cholesterol uptake (Bridgeman et al. 2022). Mechanistically, Lu et al. established that butyrate modulates E2 biosynthesis in porcine granulosa cells through cAMP-dependent signaling pathways (Lu et al. 2017). Similarly, hexanoate exerts analogous metabolic effects through lipid metabolism-related pathways. In murine models subjected to high-fat diet regimens, hexanoic acid administration significantly upregulated hepatic gluconeogenic gene expression (Ikeda et al. 2025). Our study indicates that low-yield FMD show a metabolic preference for increasing fecal SCFAs. It also revealed significant negative correlations between fecal butyrate and T, and between hexanoate and both T and E2, which were similarly associated with reduced musk secretion. Nevertheless, it should be noted that around 85 ~ 100% of ruminal SCFAs are absorbed by the epithelium, leaving little to reach the hindgut (Reynolds and Huntington 1988; Bergman 1990). Consequently, a critical point is whether the low musk yield in the FMD group stems from reduced ruminal epithelial absorption of SCFAs, resulting in their excessive accumulation in feces. Alternatively, high-yielding FMD group likely exhibit enhanced SCFA absorption, facilitating energy metabolism and supporting steroid hormone synthesis, which may ultimately enhance musk secretion. Therefore, this hypothesis requires further validation through targeted research. Nonetheless, it is evident that SCFAs contribute to the regulation of musk secretion.

Gastrointestinal microbial ecosystems, which have profound impacts on ruminant health and productivity, are influenced by diet, feeding regime, animal age, and health status (Oikonomou et al. 2013; Qiu et al. 2019b). In this investigation, we identified *Firmicutes* and *Bacteroidetes* as the dominant microbial taxa, which aligns with prior characterizations of musk deer gut microbiota where these two phyla were similarly reported as predominant components (Zhao et al. 2021; Yang et al. 2021a). In our study, the elevated abundances of *Fibrobacteres* and *Tenericutes* in the HFMD group suggest a enhanced capacity for fiber and polysaccharide degradation (Ransom-Jones et al. 2012; Wang et al. 2020). Given that fiber breakdown stimulates ruminal SCFA production, these microbial shifts likely led to an increased SCFA supply. This, in turn, supports our earlier hypothesis that rumen epithelial absorption of SCFAs may be more efficient in the HFMD group. *Verrucomicrobia* are recognized for their beneficial roles in maintaining intestinal health, modulating immune responses, and alleviating hepatic steatosis and intestinal inflammation (Cani et al. 2022; Zhang et al. 2025). In alignment with our findings, a previous study conducted in porcine rectal content also reported the presence of *Verrucomicrobia* and further identified a negative correlation between its abundance and butyrate levels(Sebastià et al. 2024). Our LEfSe analysis identified two butyrate-producing genera (*Butyrivibrio* and *Lachnoclostridium*) as being significantly enriched in the HFMD group, butyrate exhibits anti-inflammatory properties, and enhances the intestinal barrier by upregulating tight junction proteins (Mills et al. 2019). Butyrate-producing probiotics can mitigate the progression of non-alcoholic fatty liver disease (Endo et al. 2013). Of note, a recent research reported that increasing the relative abundance of the *Lachnoclostridium* genus may confer benefits for non-alcoholic fatty liver management (Dai et al. 2023). Differentially, among the biomarkers enriched in the LFMD group, *Pygmaiobacter* also can contributed to SCFAs promotion, especially butyric acid (Sun et al. 2022). *Incertae Sedis* from *Ruminococcaceae*, is integral to fermenting dietary fibers and facilitate the production of SCFAs, and increased abundance is associated with a reduced risk of liver disease-related mortality (Yamamoto et al. 2024). It can therefore be inferred from the above that butyric acid likely possesses a distinct function within this mechanism. Although not statistically significant, our high-resolution ASV level analysis detected *Incertae Sedis* (ASV_36) as a potentially enriched microbe in the HFMD group. Our ASV level analysis identified several intriguing, though not statistically significant, patterns following multiple testing correction. Specifically, multiple ASVs within the *Rikenellaceae RC9 gut group* showed suggestive enrichment in HFMD patients, with ASV_47 approaching significance and ASV_50 demonstrating a considerable effect size. These preliminary findings, while potentially limited by the sample size and stringent analytical corrections, still highlight promising microbial targets that require validation in larger independent cohorts to confirm their association with musk formation. *Rikenellaceae RC9 gut group* has been demonstrated to regulate lipid metabolism (Sebastià et al. 2024). Furthermore, correlation analysis revealed that *Ruminococcaceae UCG-014* (ASV_849) positively influences butyrate and E2 levels, suggesting a potential trend of affecting musk secretion. *Ruminococcaceae UCG-014* has been shown to promote SCFA production and exhibits a positive correlation with acetate (Liu et al. 2022) and it has been associated with hepatoprotective effects (Milton-Laskibar et al. 2022). In summary, comparative analysis of the gut microbiota in high-yield and low-yield forest musk deer using both LEfSe and MaAsLin2 revealed inconsistencies in the identification of differentially abundant microbial taxa. These discrepancies may be attributed to differences in the statistical stringency and underlying modeling principles of the two methods, as well as the limited sample size of the current study. Particularly, certain microbial signatures might only be detectable at higher resolution levels such as ASV, whereas analyses conducted at broader taxonomic classifications (e.g., genus) may mask biologically relevant but subtle variations. Nevertheless, this study offers preliminary insights into possible structural divergences in the gut microbiome between high- and low-yield FMD, providing a valuable foundation for further mechanistic investigation. Subsequently, we recommend focusing on specific bacterial groups (such as *Ruminococcaceae* and *Rikenellaceae*), and employing integrated multi-omics approaches—including metagenomics and metabolomics—in expanded cohorts to systematically unravel their functional roles in musk production.

## Conclusion

In conclusion, our study reveals distinct metabolic profiles between high- and low-yield FMD populations. Specifically, high-yield individuals demonstrate elevated steroid hormone levels in the feces, while their low-yield counterparts exhibit significantly increased concentrations of fecal butyric and hexanoic acids. Besides, we identified differential microbial biomarkers in the gut microbiota of these two populations. This suggests a potential gut microbiota–SCFA–liver–steroidogenesis–musk secretion axis, offering novel insights into the endocrine-microbial mechanisms that regulate musk secretion. To enhance musk secretion, this study recommends three key research priorities: first, to clarify how ruminal VFA absorption regulates steroid hormone synthesis in forest musk deer across physiological conditions; second, to precisely control musk secretion by targeting critical metabolic pathway components; and finally, to identify key microbial taxa involved in musk secretion—especially those linked to VFA and steroid metabolism—enabling targeted dietary strategies to improve musk yield. These efforts will provide essential theoretical and practical support for musk production under farmed conditions. Regretfully, obtaining representative tissue specimens (particularly liver or musk sac) presents significant challenges.

## Supplementary Information

Below is the link to the electronic supplementary material.

Supplementary Material 1: Figure S1. Rarefaction curve of the sequencing data; Table S1. Summary of raw and clean sequencing statistics across all samples; Table S2. Differences at the phylum level between the HFMD and LFMD groups; Table S3. Differences at the genus level between the HFMD and LFMD groups.

## Data Availability

The sequences from the current study have been deposited in the NCBI Sequence Read Archive database with the accession number PRJNA1274163.

## References

[CR1] Agus A, Clément K, Sokol H (2021) Gut microbiota-derived metabolites as central regulators in metabolic disorders. Gut 70:1174–1182. 10.1136/gutjnl-2020-323071 33272977 10.1136/gutjnl-2020-323071 PMC8108286

[CR2] Akpa MM, Point F, Sawadogo S, Radenne A, Mounier C (2010) Inhibition of insulin and T 3-Induced fatty acid synthase by hexanoate. Lipids 45:997–1009. 10.1007/s11745-010-3465-5 20811782 10.1007/s11745-010-3465-5

[CR3] Bedford A, Gong J (2018) Implications of butyrate and its derivatives for gut health and animal production. Anim Nutr 4:151–159. 10.1016/j.aninu.2017.08.010 30140754 10.1016/j.aninu.2017.08.010 PMC6104520

[CR4] Bergman EN (1990) Energy contributions of volatile fatty acids from the gastrointestinal tract in various species. Physiol Rev 70:567–590. 10.1152/physrev.1990.70.2.567 2181501 10.1152/physrev.1990.70.2.567

[CR5] Bolyen E, Rideout JR, Dillon MR, Bokulich NA, Abnet CC, Al-Ghalith GA (2019) Reproducible, interactive, scalable and extensible microbiome data science using QIIME 2. Nat Biotechnol 37:852–857. 10.1038/s41587-019-0209-9 31341288 10.1038/s41587-019-0209-9 PMC7015180

[CR6] Bridgeman S, Woo HC, Newsholme P, Mamotte C (2022) Butyrate lowers cellular cholesterol through HDAC Inhibition and impaired SREBP-2 signalling. Int J Mol Sci 23:15506. 10.3390/ijms232415506 36555149 10.3390/ijms232415506 PMC9779842

[CR7] Cani PD, Depommier C, Derrien M, Everard A, de Vos WM (2022) *Akkermansia muciniphila*: paradigm for next-generation beneficial microorganisms. Nat Rev Gastroenterol Hepatol 19:625–637. 10.1038/s41575-022-00631-9 35641786 10.1038/s41575-022-00631-9

[CR8] Chen L, Qiu Q, Jiang Y, Wang K, Lin Z, Li Z (2019) Large-scale ruminant genome sequencing provides insights into their evolution and distinct traits. Science 364:eaav6202. 10.1126/science.aav6202 31221828 10.1126/science.aav6202

[CR9] Chronister BN, Gonzalez E, Lopez-Paredes D, Suarez-Torres J, Gahagan S, Martinez D, Barros J, Jacobs DR Jr, Checkoway H, Suarez-Lopez JR (2021) Testosterone, estradiol, DHEA and cortisol in relation to anxiety and depression scores in adolescents. J Affect Disord 294:838–846. 10.1016/j.jad.2021.07.026 34375211 10.1016/j.jad.2021.07.026 PMC8992006

[CR10] Dai W, Cai D, Zhou S, Li A, Xie J, Zhang J (2023) Uncovering a causal connection between the *Lachnoclostridium* genus in fecal microbiota and non-alcoholic fatty liver disease: a two-sample Mendelian randomization analysis. Front Microbiol 14:1276790. 10.3389/fmicb.2023.1276790 38192292 10.3389/fmicb.2023.1276790 PMC10773585

[CR11] Dijkstra J, Forbes JM, France J (2005) Quantitative aspects of ruminant digestion and metabolism. CABI Publishing 1–10. 10.1079/9780851998145.0049

[CR12] Endo H, Niioka M, Kobayashi N, Tanaka M, Watanabe T (2013) Butyrate-producing probiotics reduce nonalcoholic fatty liver disease progression in rats: new insight into the probiotics for the gut-liver axis. PLoS ONE 8:e63388. 10.1371/journal.pone.0063388 23696823 10.1371/journal.pone.0063388 PMC3656030

[CR13] Escoter-Torres L, Caratti G, Mechtidou A, Tuckermann J, Uhlenhaut NH, Vettorazzi S (2019) Fighting the fire: mechanisms of inflammatory gene regulation by the glucocorticoid receptor. Front Immunol 10:1859. 10.3389/fimmu.2019.01859 31440248 10.3389/fimmu.2019.01859 PMC6693390

[CR14] Fan M, Zhang M, Shi M, Zhang T, Lei Q, Yu J, Li X, Lin S, Huang Z, Yang S, Zhou J, Li Y, Sun X, Cha M, Xu S, Liu Y, Guo X, Hu D, Liu S (2018) Sex hormones play roles in determining musk composition during the early stages of musk secretion by musk deer (*Moschus berezovskii*). Endocr J 65:1111–1120. 10.1507/endocrj.EJ18-0211 30175720 10.1507/endocrj.EJ18-0211

[CR15] Farzi A, Reichmann F, Holzer P (2015) The homeostatic role of neuropeptide Y in immune function and its impact on mood and behaviour. Acta Physiol (Oxf) 213:603–627. 10.1194/jlr.R067629 25545642 10.1111/apha.12445 PMC4353849

[CR16] Friedman H, Nylund B (1980) Intestinal fat digestion, absorption, and transport. A review. Am J Clin Nutr 33:1108–1139. 10.1111/j.1447-0578.2005.00087.x 6989228 10.1093/ajcn/33.5.1108

[CR17] Gao Z, Yin J, Zhang J, Ward RE, Martin RJ, Lefevre M, Cefalu WT, Ye J (2009) Butyrate improves insulin sensitivity and increases energy expenditure in mice, vol 58. Diabetes, pp 1509–1517. 10.2337/db08-1637 10.2337/db08-1637 PMC2699871 19366864

[CR18] Gibney J, Wolthers T, Johannsson G, Umpleby AM, Ho KKY (2005) Metabolism: growth hormone and testosterone interact positively to enhance protein and energy metabolism in hypopituitary men. Clin Trial 289:E266–E271. 10.1152/ajpendo.00483.2004 10.1152/ajpendo.00483.2004 15727949

[CR19] He Y, Wang H, Yu Z, Niu W, Qiu Q, Su H, Cao B (2018) Effects of the gender differences in cattle rumen fermentation on anaerobic fermentation of wheat straw. J Clean Prod 205:845–853. 10.1016/j.jclepro.2018.09.156

[CR20] Hu X, Liu G, Li Y, Wei Y, Lin S, Liu S, Zheng Y, Hu D (2018a) High-throughput analysis reveals seasonal variation of the gut microbiota composition within forest musk deer (*Moschus berezovskii*). Front Microbiol 9:1674. 10.3389/fmicb.2018.01674 30093891 10.3389/fmicb.2018.01674 PMC6070636

[CR21] Hu XL, Liu G, Wei YT, Wang YH, Zhang TX, Yang S, Hu DF, Liu SQ (2018b) Regional and seasonal effects on the gastrointestinal parasitism of captive forest musk deer. Acta Trop 177:1–8. 10.1016/j.actatropica.2017.09.021 28963064 10.1016/j.actatropica.2017.09.021

[CR22] Ikeda T, Nishimoto Y, Ichikawa D, Matsunaga T, Kawauchi A, Kimura I (2025) Hexanoic acid improves metabolic health in mice fed high-fat diet. BioRxiv 2:639216. 10.1101/2025.02.20.639216 10.3390/nu17172868 PMC12430857 40944255

[CR23] Le HH, Lee MT, Besler KR, Comrie JMC, Johnson EL (2022) Characterization of interactions of dietary cholesterol with the murine and human gut Microbiome. Nat Microbiol 7:1390–1403. 10.1038/s41564-022-01195-9 35982311 10.1038/s41564-022-01195-9 PMC9417993

[CR24] Li D, Chen B, Zhang L, Gaur U, Ma T, Jie H, Zhao G, Wu N, Xu Z, Xu H, Yao Y, Lian T, Fan X, Yang D, Yang M, Zhu Q, Satkoski Trask J (2016) The musk chemical composition and microbiota of Chinese forest musk deer males. Sci Rep-UK 6:18975. 10.1038/srep18975 10.1038/srep18975 PMC4705530 26744067

[CR26] Liu W, La ALTZ, Evans A, Gao S, Yu Z, Bu D, Ma L (2021) Supplementation with sodium butyrate improves growth and antioxidant function in dairy calves before weaning. J Anim Sci Biotechnol 212:1–9. 10.1186/s40104-020-00521-7 10.1186/s40104-020-00521-7 PMC7780688 33397482

[CR25] Liu K, Zhang Y, Huang G, Zheng N, Zhao S, Wang J (2022) Ruminal bacterial community is associated with the variations of total milk solid content in Holstein lactating cows. Anim Nutr 9:175–183. 10.1016/j.aninu.2021.12.005 35573096 10.1016/j.aninu.2021.12.005 PMC9079714

[CR27] Lu N, Li M, Lei H, Jiang X, Tu W, Lu Y, Xia D (2017) Biology M: Butyric acid regulates progesterone and estradiol secretion via cAMP signaling pathway in Porcine granulosa cells. J Steroid Biochem Mol Biol 172:89–97. 10.1016/j.jsbmb.2017.06.004 28602959 10.1016/j.jsbmb.2017.06.004

[CR28] Luo J, Yang H, Song BL (2020) Mechanisms and regulation of cholesterol homeostasis. Nat Rev Mol Cell Bio 21:225–245. 10.1038/s41580-019-0190-7 31848472 10.1038/s41580-019-0190-7

[CR29] Magoč T, Salzberg SL (2011) FLASH: fast length adjustment of short reads to improve genome assemblies. Bioinformatics 27:2957–2963. 10.1093/bioinformatics/btr507 21903629 10.1093/bioinformatics/btr507 PMC3198573

[CR30] Mallick H, Rahnavard A, McIver LJ, Ma S, Zhang Y, Nguyen LH, Tickle TL, Weingart G, Ren B, Schwager EH, Chatterjee S, Thompson KN, Wilkinson JE, Subramanian A, Lu Y, Waldron L, Paulson JN, Franzosa EA, Bravo HC, Huttenhower C (2021) Multivariable association discovery in population-scale meta-omics studies. PLoS Comput Biol 17:e1009442. 10.1371/journal.pcbi.1009442 34784344 10.1371/journal.pcbi.1009442 PMC8714082

[CR31] Miller WL, Auchus RJ (2001) The principles, pathways, and enzymes of human steroidogenesis. Endocr Rev 22:1616–1631. 10.1210/edrv.22.6.0452

[CR32] Mills S, Stanton C, Lane JA, Smith GJ, Ross RP (2019) Precision nutrition and the microbiome, part I: current state of the science. Nutrients 11:923. 10.3390/nu11040923 31022973 10.3390/nu11040923 PMC6520976

[CR33] Milton-Laskibar I, Cuevas-Sierra A, Portillo MP, Martínez JA (2022) Effects of Resveratrol administration in liver injury prevention as induced by an obesogenic diet: role of *Ruminococcaceae*. Biomedicines 10:1797. 10.3390/biomedicines10081797 35892696 10.3390/biomedicines10081797 PMC9330856

[CR34] Nikolaenko L, Jia Y, Wang C, Diaz-Arjonilla M, Yee JK, French SW, Liu PY, Laurel S, Chong C, Lee K, Lue Y, Lee WNP, Swerdloff RS (2014) Testosterone replacement ameliorates nonalcoholic fatty liver disease in castrated male rats. Endocrinology 155:417–428. 10.1210/en.2013-1648 24280056 10.1210/en.2013-1648 PMC5393315

[CR35] Niwińska B, Hanczakowska E, Arciszewski MB, Klebaniuk R (2017) Review: exogenous butyrate: implications for the functional development of ruminal epithelium and calf performance. Animal 11:1522–1530. 10.1017/S1751731117000167 28193308 10.1017/S1751731117000167

[CR36] Oikonomou G, Teixeira AGV, Foditsch C, Bicalho ML, Machado VS, Bicalho RC (2013) Fecal microbial diversity in pre-weaned dairy calves as described by pyrosequencing of metagenomic 16S rDNA. Associations of Faecalibacterium species with health and growth. PLoS ONE 8:e63157. 10.1371/journal.pone.0063157 23646192 10.1371/journal.pone.0063157 PMC3639981

[CR37] Oksanen J, Blanchet FG, Kindt R, Legendre P, Minchin PR, O’Hara RB, Simpson GL, Solymos P, Stevens MHH, Wagner H (2016) Vegan: community ecology package. R package version 2.3-5. R Foundation, Vienna. 10.5281/zenodo.44279

[CR38] Paradis E, Claude J, Strimmer K (2004) APE: analyses of phylogenetics and evolution in R Language. Bioinformatics 20:289–290. 10.1093/bioinformatics/btg412 14734327 10.1093/bioinformatics/btg412

[CR39] Qi WH, Lu T, Zheng CL, Jiang XM, Jie H, Zhang XY, Yue BS, Zhao GJ (2020) Distribution patterns of microsatellites and development of its marker in different genomic regions of forest musk deer genome based on high throughput sequencing. Aging 12:4445. 10.18632/aging.102895 32155132 10.18632/aging.102895 PMC7093171

[CR40] Qiu D, Xia Z, Deng J, Jiao X, Liu L, Li J (2019a) Glucorticoid-induced obesity individuals have distinct signatures of the gut microbiome. BioFactors 45:892–901. 10.1002/biof.1565 31588658 10.1002/biof.1565

[CR41] Qiu Q, Zhu Y, Qiu X, Gao C, Wang J, Wang H, He Y, Rahman MAU, Cao B, Su H (2019b) Dynamic variations in fecal bacterial community and fermentation profile of Holstein steers in response to three Stepwise density diets. Animals 9:560. 10.3390/ani9080560 31443265 10.3390/ani9080560 PMC6719243

[CR42] Quast C, Pruesse E, Yilmaz P, Gerken J, Schweer T, Yarza P, Peplies J, Glöckner FO (2013) The SILVA ribosomal RNA gene database project: improved data processing and web-based tools. Nucleic Acids Res 41:590–D596. 10.1093/nar/gks1219 10.1093/nar/gks1219 PMC3531112 23193283

[CR43] Ransom-Jones E, Jones DL, McCarthy AJ, McDonald JE (2012) The *Fibrobacteres*: an important phylum of cellulose-degrading bacteria. Microb Ecol 63:267–281. 10.1007/s00248-011-9998-1 22213055 10.1007/s00248-011-9998-1

[CR44] Reynolds CK, Huntington GB (1988) Partition of portal-drained visceral net flux in beef steers. Br J Nutr 60:539–551. 10.1079/bjn19880126 3219322 10.1079/bjn19880126

[CR45] Rial SA, Ravaut G, Malaret TB, Bergeron KF, Mounier C (2018) Hexanoic, octanoic and decanoic acids promote basal and insulin-induced phosphorylation of the Akt-mTOR axis and a balanced lipid metabolism in the HepG2 hepatoma cell line. Molecules 23:2315. 10.3390/molecules23092315 30208604 10.3390/molecules23092315 PMC6225498

[CR46] Russell N, Grossmann M (2019) Mechanisms in endocrinology: estradiol as a male hormone. Eur J Endocrinol 181. 10.1530/EJE-18-1000. R23-R43 10.1530/EJE-18-1000 31096185

[CR47] Schönfeld P, Wojtczak L (2016) Short-and medium-chain fatty acids in energy metabolism: the cellular perspective. J Lipid Res 57:943–954. 10.1194/jlr.R067629 27080715 10.1194/jlr.R067629 PMC4878196

[CR48] Sebastià C, Folch JM, Ballester M, Estellé J, Passols M, Muñoz M, García-Casco JM, Fernández AI, Castelló A, Sánchez A, Crespo-Piazuelo D (2024) Interrelation between gut microbiota, SCFA, and fatty acid composition in pigs. mSystems 9:e0104923. 10.1128/msystems.01049-23 38095419 10.1128/msystems.01049-23 PMC10804976

[CR49] Siciliano-Jones J, Murphy MR (1989) Production of volatile fatty acids in the rumen and cecum-colon of steers as affected by forage: concentrate and forage physical form. J Dairy Sci 72:485–492. 10.3168/jds.S0022-0302(89)79130-X 2703570 10.3168/jds.S0022-0302(89)79130-X

[CR50] Sun W, Cui Y, Zhang X, Wang Y, Zhang Z, Ding X, Liang H, Wang D, Sun Y, Liu S, Duan X, Lu Y, Sun T (2022) Effects of gabexate mesylate on the gut microbiota and metabolomics in rats with sepsis. J Inflamm Res 15:6581–6594. 10.2147/JIR.S392060 36506782 10.2147/JIR.S392060 PMC9733569

[CR51] Tang ZS, Liu YR, Lv Y, Duan JA, Chen SZ, Sun J, Song ZX, Wu XM, Liu L (2018) Quality markers of animal medicinal materials: correlative analysis of musk reveals distinct metabolic changes induced by multiple factors. Phytomedicine 44:258–269. 10.1016/j.phymed.2018.03.008 29551642 10.1016/j.phymed.2018.03.008

[CR52] Vandewalle J, Luypaert A, De Bosscher K, Libert C (2018) Metabolism: therapeutic mechanisms of glucocorticoids. Trends Endocrinol Metab 29:42–54. 10.1016/j.tem.2017.10.010 29162310 10.1016/j.tem.2017.10.010

[CR54] Wang Y, Huang JM, Zhou YL, Almeida A, Finn RD, Danchin A, He L (2020) Phylogenomics of expanding uncultured environmental *Tenericutes* provides insights into their pathogenicity and evolutionary relationship with *Bacilli*. BMC Genom 21:1–12. 10.1186/s12864-020-06807-4 10.1186/s12864-020-06807-4 PMC7301438 32552739

[CR53] Wang X, Wu J, Zhou X, Lv Q, Shen L, Geng S, Meng X (2024a) Relationship between aggressiveness, fecal steroid levels and musk secretion in captive alpine musk deer (*Moschus chrysogaster*). Acta Theriol Sin 44:344–350. 10.16829/j.slxb.150804

[CR55] Wang Y, Yang P, Chen T, Hu J, An X, Yao C, Xu L, Xu Y, Liu S (2024b) Analysis and comparison of blood metabolome of forest musk deer in musk secretion and non-secretion periods. Sci Rep-UK 14:16980. 10.1038/s41598-024-67981-z 10.1038/s41598-024-67981-z PMC11266552 39043795

[CR56] Wu W, Lu H, Cheng J, Geng Z, Mao S, Xue Y (2023) Undernutrition disrupts cecal microbiota and epithelium interactions, epithelial metabolism, and immune responses in a pregnant sheep model. Microbiol Spectr 11:e05320–e05322. 10.1128/spectrum.05320-22 36976022 10.1128/spectrum.05320-22 PMC10100782

[CR57] Yamamoto K, Honda T, Inukai Y, Yokoyama S, Ito T, Imai N, Ishizu Y, Nakamura M, Kawashima H (2024) Identification of the microbiome associated with prognosis in patients with chronic liver disease. Microorganisms 12:610. 10.3390/microorganisms12030610 38543661 10.3390/microorganisms12030610 PMC10974311

[CR58] Yan M, Yan Q, Yang G (2016) The mass diseases of captive musk deer. J Econ Anim 20:112–117. 10.13326/j.jea.2016.1126

[CR61] Yang Q, Meng X, Xia L, Feng Z (2003) Conservation status and causes of decline of musk deer (*Moschus* spp.) in China. Biol Conserv 109:333–342. 10.1016/S0006-3207(02)00159-3

[CR59] Yang C, Huang W, Sun Y, You L, Jin H, Sun Z (2021a) Effect of probiotics on diversity and function of gut microbiota in *Moschus berezovskii*. Arch Microbiol 203:3305–3315. 10.1007/s00203-021-02315-5 33860850 10.1007/s00203-021-02315-5

[CR60] Yang J, Peng G, Shu F, Dong D, Zheng X, Zhu C, Li X, Ma J, Pan C, Yang F, Dong W (2021b) Characteristics of steroidogenesis-related factors in the musk gland of Chinese forest musk deer (*Moschus berezovskii*). J Steroid Biochem Mol Biol 212:105916. 10.1016/j.jsbmb.2021.105916 34010686 10.1016/j.jsbmb.2021.105916

[CR62] Zhang L, Du J, Yano N, Wang H, Zhao YT, Dubielecka PM, Zhuang S, Chin YE, Qin G, Zhao TC (2017) Sodium butyrate protects against high fat diet-induced cardiac dysfunction and metabolic disorders in type II diabetic mice. J Cell Biochem 118:2395–2408. 10.1002/jcb.25902 28109123 10.1002/jcb.25902 PMC5462877

[CR64] Zhang T, Jin W, Yang S, Li Y, Zhang M, Shi M, Guo X, Li D, Zhang B, Liu S, Hu D (2021) Study of compositions of musks in different types secreted by forest musk deer (*Moschus berezovskii*). PLoS ONE 16:0245677. 10.1371/journal.pone.0245677 10.1371/journal.pone.0245677 PMC7963063 33725016

[CR63] Zhang Q, Hu WM, Deng YL, Wan JJ, Wang YJ, Jin P (2023) Dysbiosis of gut microbiota and decreased propionic acid associated with metabolic abnormality in cushing’s syndrome. Front Endocrinol (Lausanne) 13:1095438. 10.3389/fendo.2022.1095438 36755580 10.3389/fendo.2022.1095438 PMC9901362

[CR65] Zhang Y, Li Q, Tan H, Nie S (2025) Chap. 15 - *Verrucomicrobia*: *Akkermansia*. Gut Microbiota, and Health, Academic Press, PP 347–377. 10.1016/B978-0-443-21630-5.00015-0

[CR66] Zhao W, Ren Z, Luo Y, Cheng J, Wang J, Wang Y, Yang Z, Yao X, Zhong Z, Yang W, Wu X (2021) Metagenomics analysis of the gut microbiome in healthy and bacterial pneumonia forest musk deer. Genes Genom 43:43–53. 10.1007/s13258-020-01029-0 10.1007/s13258-020-01029-0 33428153

[CR69] Zhou J, Mi J, Degen AA, Ding LM, Guo XS, Shang ZH, Wang WW, Long RJ (2017) Urinary purine derivatives excretion, rumen microbial nitrogen synthesis and the efficiency of utilization of recycled Urea in Tibetan and fine-wool sheep. Anim Feed Sci Technol 227:24–31. 10.1016/j.anifeedsci.2017.03.005

[CR67] Zhou C, Zhang W, Wen Q, Bu P, Gao J, Wang G, Jin J, Song Y, Sun X, Zhang Y, Jiang X, Yu H, Peng C, Shen Y, Price Li J, Zhang X, Fan Z, Yue B (2019) Comparative genomics reveals the genetic mechanisms of musk secretion and adaptive immunity in Chinese forest musk deer. Genome Biol EVol 11:1019–1032. 10.1093/gbe/evz055 30903183 10.1093/gbe/evz055 PMC6450037

[CR68] Zhou C, Zhang Y, Qiu S, Yu H, Tu H, Wen Q, James JG, Meng Y, Wu Y, Yang N, Yue B (2020) Genomic evidence sheds light on the genetic mechanisms of musk secretion in muskrats. Int J Biol Macromol 145:1189–1198. 10.1016/j.ijbiomac.2019.10.045 31726118 10.1016/j.ijbiomac.2019.10.045

