# Supplementary material for: Fecal steroids, short-chain fatty acids, and microbiota in high- versus low-yielding forest musk deer
Source: AMB Express. 2025 Nov 10;15:168. doi: 10.1186/s13568-025-01967-6 (PMC12602856; doi:10.1186/s13568-025-01967-6)
Supplement: Supplementary file 1 — Supplementary Material 1: Figure S1. Rarefaction curve of the sequencing data; Table S1. Summary of raw and clean sequencing statistics across all samples; Table S2. Differences at the phylum level between the HFMD and LFMD groups; Table S3. Differences at the genus level between the HFMD and LFMD groups. [file 13568_2025_1967_MOESM1_ESM.docx]

**Supplementary information**

**Table S1** Summary of raw and clean sequencing statistics across all samples.

| Samples | Raw tags | Raw avg. length | Raw Q20 | Raw Q30 | Clean tags | Clean avg. length | Clean Q20 | Clean Q30 | Efficiency rate |
| --- | --- | --- | --- | --- | --- | --- | --- | --- | --- |
| HFMD-1 | 57715 | 291.9 | 99.47 | 98.20 | 55840 | 291.5 | 99.6 | 98.6 | 96.75 |
| HFMD-2 | 52843 | 291.9 | 99.46 | 98.16 | 51207 | 291.5 | 99.59 | 98.55 | 96.90 |
| HFMD-3 | 57147 | 291.9 | 99.42 | 97.99 | 55092 | 291.5 | 99.56 | 98.45 | 96.40 |
| HFMD-4 | 56650 | 291.8 | 99.46 | 98.12 | 54845 | 291.4 | 99.59 | 98.52 | 96.81 |
| HFMD-5 | 55797 | 291.9 | 99.34 | 97.86 | 53584 | 291.5 | 99.48 | 98.30 | 96.03 |
| HFMD-6 | 59753 | 291.8 | 99.39 | 97.9 | 57432 | 291.4 | 99.55 | 98.38 | 96.12 |
| HFMD-7 | 53715 | 291.9 | 99.49 | 98.28 | 52066 | 291.6 | 99.62 | 98.67 | 96.93 |
| LFMD-1 | 50429 | 291.9 | 99.48 | 98.28 | 48851 | 291.6 | 99.61 | 98.65 | 96.87 |
| LFMD-2 | 51220 | 291.8 | 99.44 | 98.15 | 49287 | 291.5 | 99.57 | 98.57 | 96.23 |
| LFMD-3 | 57714 | 291.9 | 99.42 | 98.02 | 55511 | 291.6 | 99.56 | 98.46 | 96.18 |
| LFMD-4 | 50933 | 291.9 | 99.41 | 98.00 | 48967 | 291.5 | 99.56 | 98.45 | 96.14 |
| LFMD-5 | 58906 | 291.9 | 99.47 | 98.23 | 57015 | 291.6 | 99.6 | 98.61 | 96.79 |
| LFMD-6 | 52600 | 291.8 | 99.43 | 98.08 | 50794 | 291.4 | 99.57 | 98.50 | 96.57 |
| LFMD-7 | 54409 | 291.9 | 99.44 | 98.07 | 52738 | 291.5 | 99.57 | 98.47 | 96.93 |

**
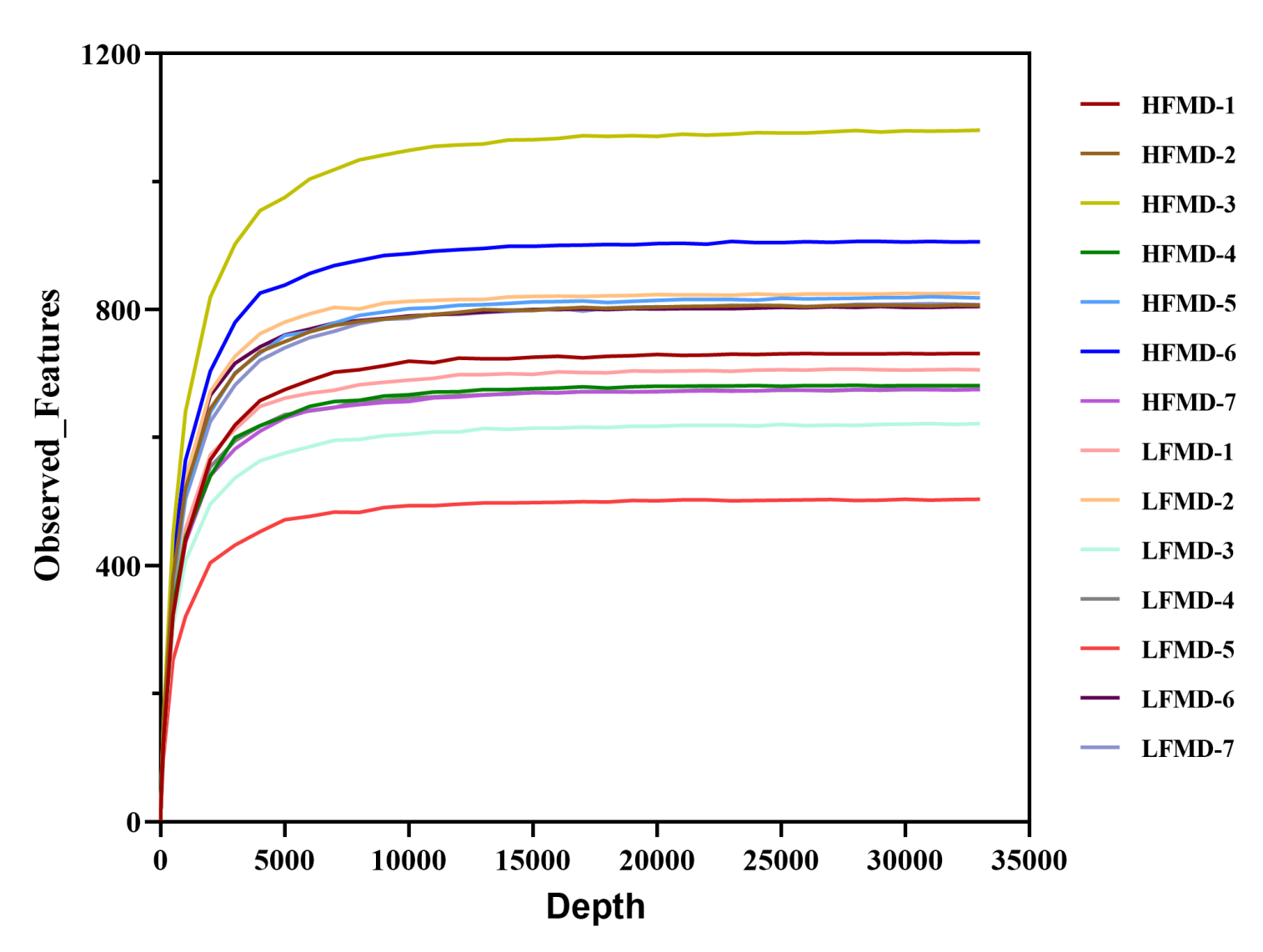
**

**Figure S1** Rarefaction curve of the sequencing data

**Table S2** Differences at the phylum level between the HFMD and LFMD groups.

| Phylum | HFMD | LFMD | SEM | *p* value | FDR |
| --- | --- | --- | --- | --- | --- |
| *Firmicutes* | 61.38 | 64.11 | 2.095 | 0.984 | 0.984 |
| *Bacteroidetes* | 32.69 | 28.49 | 2.153 | 0.461 | 0.691 |
| *Planctomycetes* | 1.85 | 4.01 | 0.703 | 0.091 | 0.363 |
| *Euryarchaeota* | 0.59 | 1.05 | 0.193 | 0.151 | 0.519 |
| *Proteobacteria* | 0.34 | 0.46 | 0.058 | 0.206 | 0.617 |
| *Verrucomicrobia* | 0.39 | 0.17 | 0.080 | 0.037 | 0.223 |
| *Actinobacteria* | 0.29 | 0.37 | 0.113 | 0.814 | 0.955 |
| *Fibrobacteres* | 0.41 | 0.02 | 0.114 | 0.013 | 0.107 |
| *Cyanobacteria* | 0.15 | 0.32 | 0.065 | 0.284 | 0.668 |
| *Tenericutes* | 0.26 | 0.16 | 0.027 | 0.013 | 0.107 |
| *Kiritimatiellaeota* | 0.27 | 0.02 | 0.131 | 0.427 | 0.691 |
| F：B | 1.99 | 2.73 | 0.368 | 0.585 | 0.780 |

F:B = *Firmicutes*/*Bacteroidetes* rati

**Table S3** Differences at the genus level between the HFMD and LFMD groups.

| Genus | HFMD | LFMD | SEM | *p* value | FDR |
| --- | --- | --- | --- | --- | --- |
| *Bacteroides* | 11.37 | 13.46 | 1.788 | 0.995 | 0.997 |
| *Ruminococcaceae UCG-005* | 9.84 | 10.91 | 1.070 | 0.685 | 0.973 |
| *Christensenellaceae R-7 group* | 8.61 | 9.25 | 0.815 | 0.874 | 0.997 |
| *Rikenellaceae RC9 gut group* | 7.94 | 6.31 | 0.796 | 0.499 | 0.878 |
| *Ruminococcaceae UCG-010* | 6.48 | 5.89 | 0.491 | 0.468 | 0.878 |
| Unclassified *Lachnospiraceae* | 4.46 | 4.46 | 0.480 | 0.805 | 0.997 |
| *Prevotellaceae UCG-004* | 3.99 | 2.30 | 0.584 | 0.446 | 0.878 |
| *Lachnospiraceae NK4A136 group* | 2.74 | 3.08 | 0.409 | 0.915 | 0.997 |
| *[Eubacterium] coprostanoligenes group* | 2.58 | 2.94 | 0.379 | 0.986 | 0.997 |
| *p-1088-a5 gut group* | 1.68 | 3.76 | 0.694 | 0.101 | 0.743 |
| *Ruminococcaceae UCG-014* | 2.90 | 1.95 | 0.299 | 0.088 | 0.743 |
| *Lachnospiraceae AC2044 group* | 2.48 | 1.97 | 0.278 | 0.933 | 0.997 |
| *Alistipes* | 2.00 | 1.67 | 0.251 | 0.641 | 0.959 |
| *Ruminococcaceae UCG-013* | 1.38 | 1.95 | 0.267 | 0.454 | 0.878 |
| *Marvinbryantia* | 1.47 | 2.04 | 0.466 | 0.366 | 0.878 |
| Unclassified *Muribaculaceae* | 1.58 | 0.90 | 0.420 | 0.237 | 0.878 |
| Unclassified *Ruminococcaceae* | 1.19 | 1.26 | 0.129 | 0.941 | 0.997 |
| *Roseburia* | 1.43 | 1.03 | 0.121 | 0.346 | 0.878 |
| *Prevotella 1* | 2.19 | 0.24 | 0.926 | 0.007 | 0.316 |
| *Phascolarctobacterium* | 1.21 | 1.09 | 0.142 | 0.279 | 0.878 |
| *dgA-11 gut group* | 0.89 | 1.12 | 0.263 | 0.186 | 0.878 |
| *Tyzzerella 4* | 0.95 | 1.09 | 0.137 | 0.952 | 0.997 |
